# The health perceptions, dengue knowledge and control willingness among Dai ethnic minority in Yunnan Province, China

**DOI:** 10.1186/s12889-021-11864-9

**Published:** 2021-10-12

**Authors:** Hui Liu, Cheng-Jiang Fang, Jian-Wei Xu

**Affiliations:** 1 Yunnan Institute of Parasitic Diseases, Yunnan Provincial Key Laboratory of Vector-borne Diseases Control and Research, Xiyuan Road, Pu’er City, 665000 China; 2 Institute of Pathogens and Vectors, Basic Medical College, Dali University, Wanhua Road, Xiaguang District, Dali City, 671000 China; 3 Pu’er Blood Bank, Chayuan Road, Pu’er City, 665000 Yunnan Province China

**Keywords:** Health perception, Dengue fever, Knowledge, Willingness, Dai ethnic minority, China

## Abstract

**Background:**

Outbreaks of dengue fever are often found among Dai ethnical communities along China-Myanmar border. The objective of this study was: 1) to investigate residents’ health perceptions, knowledge and control willingness to participate in dengue control and 2) to identify factors associated with control willingness among the Dai ethnic community.

**Methods:**

This is a mixed method study of a cross-sectional design, in which qualitative in-depth interviews and quantitative household questionnaire surveys are included.

**Results:**

Questionnaire was administered to 261 household heads, and in-depth interview was conducted with 18 key informants. Of them, many participants (70%, 182/259) and 12 key informants (66.7%) from the two rural communities believed that the Lord Buddha would protect the good people. Majority of the participants (81.4%, 206/253) knew that fever was one of dengue fever symptoms and most of them (82.2%, 213/259) indicated that mosquitoes could transmit dengue fever. However, only one third of the participants (30.1%, 78/259) indicated the perceived susceptibility of dengue fever, and only a half of them (50.2%, 130/259) indicated the perceived severity of dengue fever. Multivariate logistic analysis (MLA) indicated that the participants with family wealth index (FWI) 4–5 (OR: 22.9728; 95%CI: 2.4257–217.5688, *p* = 0.0063) were more likely to turn containers upside down (TCUD) compared to those with FWI 1–3; and the participants in the urban community (OR: 0.0239; 95%CI: 0.0019–0.3032, *p* = 0.004) were less likely to TCUD compared to those in the two rural communities. Around one third of the participants (36.8%, 96/239) reported that they were willing to seek treatment first for dengue fever from public health facilities. The MLA identified that the participants with the perceived severity of dengue fever (OR: 5.0564; 95%CI: 2.0672–12.3683, *p* = 0.0004), and with beliefs of sound hygiene helpful to people’s health (OR: 11.5671; 95%CI: 2.0505–65.2502, *p* = 0.0055) were more likely to seek treatment first for dengue fever from the public health facilities.

**Conclusion:**

The study finds that most of Dai people have sound knowledge. However, health educational interventions should target to promote the perceived susceptibility and the perceived severity of dengue fever among Dai people.

**Supplementary Information:**

The online version contains supplementary material available at 10.1186/s12889-021-11864-9.

## Background

Dengue fever is one of the hardest-to-control arboviral diseases in the world. The World Health Organization (WHO) estimated 284–528 million dengue infections yearly in 128 territories [1, 2]. Dengue also causes a substantial economic burden to governments and households [2, 3]. In China, dengue fever incidence and areas affected have steadily increased since 2000 [4, 5]. Dengue has become one of the main public health problems, while malaria was successfully controlled along China-Myanmar border [6, 7]. The Dai people in Yunnan Province, Southwestern China, are one of the most affected populations by dengue fever outbreaks in China [8].

There are no effective antiviral therapies currently available for dengue fever and to date the only licensed dengue vaccine is not fully protectable [9]. Thus intensified vector control is one of the most widely used strategies to prevent dengue virus transmission [10, 11]. Water containers and discarded tires left by local people are primary productive habitats of *Aedes sp* mosquitoes. Clearance of the habitats can reduce density of *Aedes sp* mosquitoes and further to reduce dengue virus transmission [10]. Community involvement is one of effective strategies to manage the habitats of *Aedes sp* mosquitoes [11, 12]. Early diagnosis is very important for effectively clinical management to improve patient prognosis and to reduce transmission of dengue virus [13, 14]. In China, laboratory test for dengue fever is only available in public health facilities, thus that suspected dengue fever patients seek appropriate treatment first from public health facilities is helpful to detection of dengue fever [6].

Local people’s health beliefs and knowledge of dengue fever may likely influence their willingness to participate in dengue fever control and then further influence transmission of dengue virus [14, 15]. The health beliefs are generally shaped by people’s expectancies about the environmental cues that are about how events are connected - about what leads to what, the consequences of their own actions that are about how individual behaviors are likely to influence outcomes, and their own competence to perform the behavior needed to influence outcomes. The basic concepts of the Health Belief Model (HBM) are health knowledge, health concerns (perceived susceptibility and perceived severity), perceived cost-benefits, perceived barriers to taking health action, cue to action and self-efficacy [16–18]. Chinese Government has put a highly political will and commitment to dengue control, and also increased investment in researches on biomedicine, surveillance system and public responses since the epidemic of dengue fever in Guangdong Province [12]. Nevertheless, the HBM study has rarely been applied in an attempt to understand social determinants of dengue control among the ethnic minorities in China.

This study was carried out among three Dai ethnic communities from December 2017 to April 2018, prior to the local dengue transmission season. The objective of this study was: 1) to investigate residents’ health beliefs, knowledge and willingness to participate in dengue fever control and 2) to identify factors associated with the control willingness among the Dai ethnic community.

## Methods

### Study design and setting

**Fig. 1:**
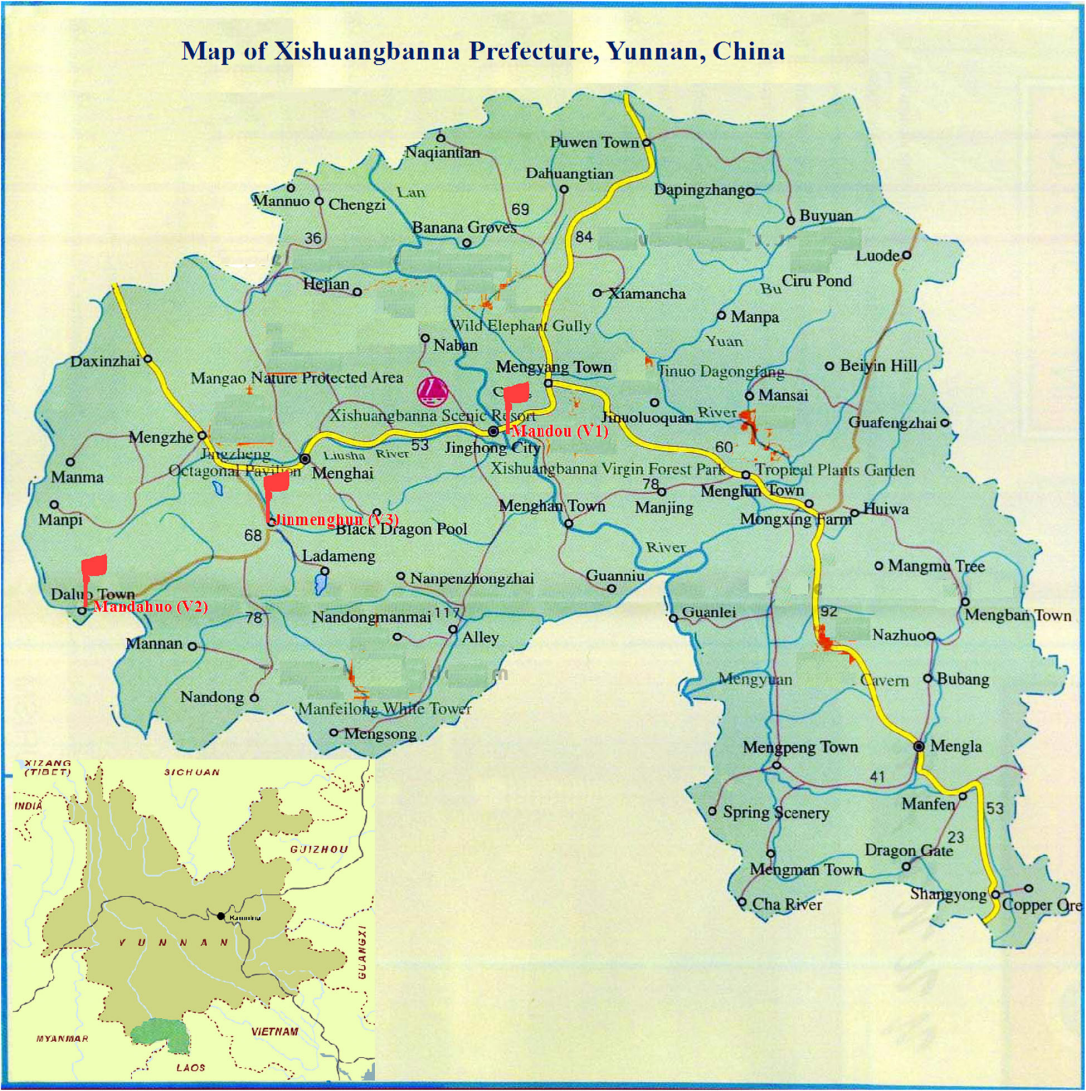
Location of study site: The red flags indicate the study site in Xishuangbanna Prefecture, Yunnan, China. The figure was generated by using the drawing tool of Microsoft Windows 10 software based on original Yunnan map and Xishuangbanna map collected in the Map Bank of Yunnan Institute of Parasitic Diseases (YIPD). The bank is open access and use to YIPD’s staff without any charges or barriers

This is a mixed method study of a cross-sectional design, in which qualitative in-depth interviews and quantitative household questionnaire surveys were included. For this study, three villages with high, middle and low incidences of dengue fever in 2017 were sampled from Xishuangbanna Prefecture, Yunnan Province (Fig. 1). A total of 18 key informants (six per village) were sampled for qualitative in-depth interviews. Based on an estimated 20% of adult people who had knowledge that dengue virus is transmitted by mosquitoes, standard value normal distribution at 95% confidence levels and 5% precision, a sample size of at least 250 household heads for quantitative household questionnaire survey was determined [19]. Assuming a population with higher incidence of a disease may be more knowledgable about the disease, the crude sampling ratio was set up around 1:2:2 for the three communities experienced high, middle and low incidences of dengue fever in 2017.

Xishuangbanna Prefecture has three counties (i.e., Menghai, Jinghong and Mengla) with a total population of 1.2 million, including 350 thousand of Dai people. Dai people, one of the main ethnicities in the Greater Mekong Subregion, are also known as Shan in Myanmar, Thai Yai in Thailand and Lao in Lao PDR. All the participants of this study were Dai people from the three communities with high, middle and low incidences of dengue fever in 2017. Manduo is an urbanized community with a population of 889 and with the high incidence of dengue fever in 2017 on the north bank of the Lancangjiang River (the upper section of Mekong River) in Jinghong City. Mandahuo is a village with a population of 675 and with the middle incidence of dengue fever in 2017 on the China-Myanmar border, Daluo Town, Menghai County. Jingmenghun is a rural community with a population of 879 and with only one imported dengue fever case in 2017 in Menghun town, Menghai County (Fig. 1). Manduo and Mandahuo are suitable for reproduction of mosquitoes and transmission of vector-borne diseases due to their lower attitude (around 600 m), tropical climate and adequate precipitation. The key informants estimated that about 20% of people in Mandou had dengue fever episodes, and 1.5% of people in Mandahuo had dengue fever episodes in 2017, respectively. Jingmenghun is less appropriate for reproduction of mosquitoes and transmission of vector-borne diseases because of its altitude of 1198 m and cool climate.

### In-depth interviews

The qualitative in-depth interview guideline (Additional file 1) covered: 1) local health problems, people’s beliefs related to religions, causes of diseases and perceived local dengue situation; 2) dengue knowledge consisted of symptoms of dengue fever, morphological characteristics of *Aedes spp*, breeding sites and biting time of *Aedes spp*; and 3) willingness to participate dengue control, i.e. management *of Aedes spp* breeding sites and seeking treatment first for dengue fever from public health facilities. The qualitative in-depth interviews were conducted with six key informants at each community (a total of 18 key informants, three communities), including one health worker, three village leaders and two community representatives who were selected by the other villagers. All of the key informants can understand and speak mandarin. Two investigators worked together to conduct every interview, one investigator discussed with the interviewees, and another took notes in Chinese.

### Household questionnaire survey

A household was a sampling unit that was defined as all people eating from the same cooking pot. The quantitative household questionnaire (Additional file 2) consisted of socio-demographic characteristics, health beliefs, dengue fever knowledge, willingness to participate dengue control and household’s physical assets. Where the health beliefs consisted of nine items, the dengue fever knowledge consisted of 26 items and the willingness consisted of 13 items. The household’s physical assets, including housing, walls, roofs, transportation tools and family belongings, were used to categorize family wealth index (FWI) into five levels (Table 1). The simple random sampling was used to select households based on existing household numbers from local authorities for this study. All participants can understand and speak Mandarin, thus the surveys were conducted in Mandarin. Investigators first visited each sampled household and introduced to the household head about this survey’s purpose and answered questions raised. Following an oral informed consent obtained, the questionnaire was administered to the household head.

**Table 1:** Principal components for construction of the family wealth index (FWI)

Family wealth index	Housing characteristics	Transportation tools	Family belongings
**1 Most poor**	Bamboo walls and sheet iron roofs	None	None or chickens
**2 Mid low**	Wood walls and sheet iron roofs	Bicycles	Pigs or goats
**3 Middle**	Brick walls, wood girders and terracotta roofs	Motorcycles	Cattle or horses
**4 Mid high**	Brick concrete walls and terracotta roofs	Tractors	TV sets or refrigerators
**5 Least poor**	Steel and concrete	Cars	Shops

### Statistical analysis

Both qualitative and quantitative data were entered with the Microsoft Excel 2007. The transcriptions of qualitative in-depth interviews were coded according to topics and then entered into cells in Microsoft Office Excel 2007. The same content records were combined by code sequencing. Two researchers generated themes independently, and then they compared and discussed their findings to synthesize findings under the same spectrums to finalize the health themes [8, 14]. The health themes identified from the qualitative study were independently presented first closely following the respondents’ characteristics. To synthesize the information of qualitative study with the results of quantitative data analysis well, the more detailed results of qualitative study are interspersed with the quantitative data in each part of the results.

All statistical analyses of the quantitative data were performed with the Epi Info 7.2 (Centers for Disease Control and Prevention, USA). Non-response and irrelevant answers were treated as missing values and therefore excluded from the analyses. The proportion and its 95% confidence interval (CI) were calculated for each item of health beliefs, knowledge, prevention and treatment-seeking willingness. The proportions of each item were compared by chi-squared Fisher’s exact test across the three study sites. Values of *p* < 0.05 were considered statistically significant.

A multivariate logistic analysis (MLA) was used to identify factors associated with control willingness. The outcome “1″ in the MLA model means that a household’s willingness of turning containers upside down, and seeking treatment first from public facilities if one of the family members had suspected symptoms of dengue fever. In modeling strategy, the independent variables of univariate logistic analysis were items with values of *P* < 0.05 in the chi-squared Fisher’s exact test across the three study sites. In addition to this criterion, two counties (Jinghong and Menghai) where the three study sites are located were included in the univariate logistic analysis in considering the difference of the dengue fever incidence and the urbanization degree of the two counties. The independent variables were included in the MLA model if they had a value of *p* < 0.25 on the univariate logistic analysis.

### Concept definition

Local people’s health beliefs can influence their knowledge and control willingness. Health belief here was defined as the general perception of elements relevant to health and disease and not limited to unique beliefs about dengue fever. It shows local perception of the effect of socioeconomic, natural and religious elements on their health and their perception of disease causes. In addition, respondent’s knowledge regarding dengue includes their knowledge of clinical symptoms, vectors, prevention, treatment and perceived severity of dengue fever [8]. Control willingness is defined as a participant’s attitude about participating in community-based vector control like cleaning dumps and turn containers upside down, and seeking treatment first from public health facilities if one of the family members had suspected symptoms of dengue fever [14].

## Results

### Participants’ characteristics

A total of 18 key informants with age range 30 to 57 years old were interviewed in depth. There were more males than females (10 vs. 8) for the key informants. A total of 261 valid questionnaires were completed. As Table 2 shows, the mean age of the sample was 43.4 years old (median: 41.0, range: 17–84), and the mean age of the participants in the urban community (39.2 years) was younger compared to those in the two rural communities (45.8 years in Mandahuo and 43.6 years in Jingmenhun). There were fewer males (36.8%, 96/261) than females among participants of the quantitative study. About a half of participants (49.4%, 129/261) had formal school education for less than 6 years. Most of the participants (98.8%, 256 /259) of the quantitative study and the key informants (94.4%, 17/18) of the qualitativ**e** study were Buddhists. Most of participating households (95%, 246/259) were not poor with a family wealth index (FWI) 4–5.

**Table 2:** Demographic characteristics, health perceptions, dengue knowledge and control willingness (*N* = 261)

Variables	Total No. (%, 95CI^a^), ***n*** = 261	No. (%, 95CI) in Manduo, ***n*** = 61	No. (%, 95CI^a^) in Mandahuo, ***n*** = 100	No. ([%, 95CI^a^) in Jingmenghun, ***n*** = 100	***P***-value
**Demographics**					
**Male sex of household heads**	96(36.8, 30.9–43.0)	25(41.0, 28.6–54.3)	27(27.0, 18.6–36.8)	44(44.0, 34.1–54.3)	0.0331
**Age of household heads (years)**					
**18–40**	124(47.5, 41.3–53.8)	39(63.9, 50.6–75.8)	42(42.0, 32.2–52.3)	43(43.0, 33.1–53.3)	0.0134
**41–84**	137(52.5, 46.2–58.7)	22(36.1, 24.2–49.4)	58(58.0, 47.7–67.8)	57(57.0, 46.7–66.9)	0.0134
**School education (years)**	43				
**≤ 6**	129(49.4, 87.2–55.7)	13(21.3, 11.9–33.7)	36(36.0, 26.6–46.2)	80(80.0, 70.8–87.3)	< 0.0001
**7–9**	58(22.2, 17.3–27.8)	30(49.2, 36.1–62.3)	12(12.0, 6.4–20.0)	16(16.0, 9.4–24.7)	< 0.0001
**> 9**	74(28.4, 23.0–34.2)	18(29.5, 18.5–42.6)	52(52. 0, 41.8–62.1)	4(4.0, 1.1.9–9.9)	< 0.0001
**Family wealth index**	*n* = 259	*n* = 59	*n* = 100	*n* = 100	
**1 Most poor**	10(3.9, 0–7.0)	1(1.7, 0–9.1)	8(8.0, 3.5–15.2)	1(1.0, 0–5.5)	0.0220
**2 Mid low**	0(0, 0–1.4)	0(0, 0–0.6)	0(0, 0–3.6)	0(0, 0–3.6)	–
**3 Middle**	3(1.2, 0.2–3.4)	2(3.4, 0.4–11.7)	1(1.0, 0–5.5)	0(0, 0–3.6)	–
**4 Mid high**	82(31.7, 26.0–37.7)	7(11.9, 4.9–22.9)	74(74.0, 64.3–82.3)	1(1.0, 0–5.5)	< 0.0001
**5 Least poor**	164(63.3, 57.1–69.2)	49(83.3, 71.0–91.6)	17(17.0, 10.2–25.8)	98(98.0, 93.0–99.7)	< 0.0001
**Health beliefs in general**					
**Poverty is a cause of ill health**	*n* = 259	*n* = 60	*n* = 99	*n* = 100	
	77(29.7, 24.2–35.7)	7(11.7, 4.8–22.6)	16 (16.2, 9.5–24.9)	54(54.0, 43.7–64.0)	< 0.0001
**People with evil practices may be punished by diseases**	*n* = 260	*n* = 60	*n* = 100	*n* = 100	
	95(36.5, 30.7–42.7)	3(5.0, 1.0–13.9)	16(16.0, 9.4–24.7)	76(76.0, 66.4–84.0)	< 0.0001
**The Lord Buddha will protect good people**	182(70.0, 64.0–75.5)	3(5.0, 1.0–13.9)	99(99.0, 94.6–100.0)	80(80.0, 70.8–87.3)	< 0.0001
**All natural factors influence health**	*n* = 259	*n* = 60	*n* = 100	*n* = 99	
	198(76.5, 70.8–81.5)	59(98.3, 91.1–100.0)	40 (40.0, 30.3–50.3)	99(100.0, 100.0–100.0)	< 0.0001
**Beliefs on environments associated with diseases**	*n* = 258	*n* = 59	*n* = 99	*n* = 100	
**Too hot**	120(46.5, 40.3–52.8)	14(23.7, 13.6–36.6)	9(9.1, 4.2–16.6)	97(97.0, 91.5–99.4)	< 0.0001
**Too cold**	186(72.1, 66.2–77.5)	33(55.9, 42.4–68.8)	68(68.7, 58.6–77.6)	85(85.0, 76.5–91.4)	0.0003
**Too rainy**	119(46.2, 39.9–52.4)	5(8.5, 2.8–18.7)	36(36.4, 26.9–46.6)	78(78.0, 68.6–85.7)	< 0.0001
**Too forested**	22(8.5, 5.4–12.6)	0(0, 0–6.1)	0(0, 0–3.6)	22(22.0, 14.3–31.4)	–
**Rivers, streams and clear water pools**	29(11.2, 7.7–15.7)	4(6.8, 1.9–16.5)	0(0, 0–3.6)	25(25.0, 16.9–34.6)	–
**Polluted water**	74(28.7, 23.2–34.6)	25(42.4, 29.6–55.9)	4(4.0, 1.1–10.0)	45(45.0, 35.0–55.3)	< 0.0001
**Poor hygiene**	220(85.3, 80.3–89.4)	59(100, 93.9–100.0)	80(80.8, 71.7–88.0)	81(81.0, 71.9–88.2)	< 0.0001
**Beliefs on environment benefiting health**	*n* = 260	*n* = 60	*n* = 100	*n* = 100	
**Clean and sound hygiene**	213(81.9, 76.7–86.4)	34(56.7, 43.2–69.4)	79(79.0, 69.7–86.6)	100(100.0, 100.0–100.0)	< 0.0001
**No polluted water**	100(38.5, 32.5–44.7)	16(27.7, 16.1–39.6)	8(8.0, 3.5–15.2)	76(76.0, 66.4–84.0)	< 0.0001
**Many flowers, grass and trees**	110(42.3, 36.2–48.6)	48(80.0, 67.7–89.2)	0(0, 0–3.6)	62(62.0, 51.7–71.5)	< 0.0001
**Good hygiene can reduce diseases**	210(80.9, 75.5–85.4)	25(41.7, 29.1–55.1)	96(96.0, 90.1–98.9)	89(89.0, 81.2–94.4)	< 0.0001
**Perceived severity**	*n* = 259	*n* = 60	*n* = 100	*n* = 99	
**Easy to contract dengue**	78(30.1, 24.6–36.1)	14(23.3, 13.4–36.0)	19(19.0, 11.8–28.1)	45(45.5, 35.4–55.8)	0.0001
**Not easy to get dengue**	160(61.8, 55.6–67.7)	46(76.7, 64.0–86.6)	66(66.0, 55.8–75.2)	48(48.5, 38. 3–58.8)	0.0010
**A severe illness**	130(50.2, 43.9–56.4)	16(26.2, 15.8–39.1)	62(62.0, 51.8–71.5)	52(53.1, 42.7–63.2)	0.0001
**A deadly disease**	102(39.4, 33.4–45.6)	7(11.7, 4.8–22.6)	60(60.0, 49.7–69.7)	35(35.4, 26.0–45.6)	< 0.0001
**Do not know**	21 (8.1, 5.1–12.1)	0(0, 0–6.0)	15(15.0, 8.7–23.5)	6(6.1, 2.3–12.7)	0.0022
**Transmissibility**	198(75.9, 70.2–80.9)	60(100.0, 94.0–100.0)	62(62.0, 51.8–71.5)	76(76.0, 66.4–84.0)	< 0.0001
**Directly transmittable from person to person**	*N* = 256	*N* = 61	*n* = 99	*N* = 96	
	36(14.1, 10.1–18.9)	2(3.3, 0.4–11.4)	5(5.1, 1.7–11.4)	29 (30.2, 21.3–40.4)	< 0.0001
**Heard about dengue**	231(88.5, 84.0–92.1)	60(100.0, 94.0–100.0)	84(84.0, 75.3–90.6)	87(87.0, 78.8–92.9)	0.0060
**Knowledge of dengue symptoms**	*n* = 253	*n* = 61	*n* = 100	*n* = 92	
**Fever**	206(81.4, 76.0–85.9)	61(100.0, 94.1–100.0)	73(73.0, 63.2–81.4)	72(78.2, 68.4–82.2)	0.0001
**Headache**	177(70.0, 63.9–75.5)	60(98.4, 91.2–100.0)	38(38.0, 28.5–48.3)	79(85.9, 77.0–92.3)	< 0.0001
**Orbital pain**	65(25.7, 20.4–31.5)	18(29.5, 18.5–42.6)	14(14.0, 7.9–22.4)	33 (35.9, 26.1–46.5)	0.0016
**Pantalgia**	140(55.3, 48.9–61.6)	31(50.8, 37.7–63.9)	47(47.0, 36.9–57.2)	62(67.4, 56.8–76.8)	0. 0093
**Rash**	49(19.4, 14.7–24.8)	16(26.2, 15.8–39.1)	11(11.0, 5.6–18.8)	22(23.9, 15.6–33.9)	0.0219
**Others**	29(11.5, 7.8–16.0)	0(0, 0–5.9)	28(28.0, 19.5–37.9)	1(1.1, 0.02–5.9)	< 0.0001
**Not know or no response**	8(3.2, 1.4–6.1)	0(0, 0–5.9)	0(0, 0–3.6)	8(8.7, 3.8–16.4)	0.0007
**Knowledge of dengue causes**	*n* = 259	*n* = 61	*n* = 100	*n* = 98	
**Bacteria**	0(0, 0–1.8)	0(0, 0–5.9)	0(0, 0–3.6)	0(0, 0–4.7)	–
**Viruses**	9(3.5, 1.6–6.5)	7(11.5, 4.7–22.2)	1(1.0, 0.03–5.4)	1(1.0, 0.03–5.6)	0.0005
**Mosquitoes**	213(82.2, 77.0–86.7)	60(98.4, 91.2–100.0)	70(70.0,60.0–78.8)	83(84.7, 74.2–89.8)	< 0.0001
**Eat improper or dirty food**	5(1.9, 0.6–4.4)	0(0, 0–5.9)	0(0, 0–3.6)	5(5.1, 1.7–11.5)	0.0152
**Animals**	2(0.8, 0.1–2.8)	1(1.6, 0.04–8.8)	0(0, 0–3.6)	1(1.0, 0.03–5.6)	–
**Flies**	0(0, 0–1.8)	0(0, 0–5.9)	0(0, 0–3.6)	0(0, 0–4.7)	–
**Be rained or shower with cold water**	0(0, 0–1.8)	0(0, 0–5.9)	2(0, 0–3.6)	0(0, 0–4.7)	–
**Others**	0(0, 0–5.9)	0(0, 0–5.9)	1(1.0, 0.03–5.4)	1(1.0, 0.03–5.6)	–
**Not know or no response**	36(13.9, 9.9–18.7)	0(0, 0–5.9)	25(25.0, 16.9–34.7)	11(11.2, 5.7–19.2)	< 0.0001
**Knowledge of dengue mosquitoes**					
**Piebald or** ***Aedes spp***	*n* = 255	*n* = 60	*n* = 97	*n* = 98	
	194(76.1, 70.4–81.2)	59(98.3, 91.1–100.0)	41(42.3, 32.3–52.7)	94(95.9, 89.9–98.9)	< 0.0001
**Biting time**	*n* = 261	*n* = 61	*n* = 100	*n* = 100	
**24 h**	114(43.7, 37.6–49.9)	56(91.8, 81.9–97.3)	25(25.0, 16.9–34.7)	34(34.0, 24.8–44.2)	< 0.0001
**Daytime**	117(44.8, 38.7–51.1)	5(8.2, 2.7–18.1)	54(54.0, 43.7–64.0)	58(58.0, 47.7–67.8)	< 0.0001
**Night**	17(6.5, 3.8–10.2)	0(0, 0–6.0)	17(17.0, 10.2–25.8)	0(0, 0–3.6)	< 0.0001
**Not know or no response**	14(5.4, 3.0–8.8)	0(0, 0–6.0)	6(6.0, 2.2–12.6)	8(8.0, 3.5–15.2)	0.0860
***Aedes*** **larvae habitats**					
**All water sites**	*n* = 260	*n* = 61	*n* = 99	*n* = 100	
	226(86.9, 82.2–90.8)	60(98.4, 91.2–100.0)	68(68.7, 58.6–77.6)	98(98.0, 93.0–99.8)	< 0.0001
**Watered containers or small-scale ponds**	*n* = 247	*n* = 50	*n* = 97	*n* = 100	
	220(89.1, 84.5–92.7)	50(100.0, 100.0–100.0)	70(72.2, 62.1–80.8)	100(100.0, 100.0–100.0)	< 0.0001
**Knowledge regarding reducing** ***Aedes*** **breeding sites**	*n* = 261	*n* = 61	*n* = 100	*n* = 100	
**Maintain sound hygiene**	122(46.7, 40.6–53.0)	29(47.5, 34.6–60.7)	13 (13.0, 7.1–21.2)	80 (80.0, 70.8–87.3)	< 0.0001
**Turn containers upside down**	242(92.7, 88.9–95.6)	61(100.0, 100.0–100.0)	81(81.0, 71.9–88.2)	100(100.0, 100.0–100.0)	< 0.0001
**Drain watered small-scale ponds**	69 (26.4, 21.2–32.2)	9(14.8, 7.0–26.2)	5(5.0, 3.6–11.3)	55(55.0, 44.7–65.0)	< 0.0001
**Knowledge of preventing** ***Aedes*** **bites**	*n* = 261	*n* = 61	*n* = 100	*n* = 100	
**Door and window screens**	120(45.9, 39.8–52.2)	21(34.4, 22.7–47.7)	10 (10.0, 4.9–17.6)	89(89.0, 81.2–94.4)	< 0.0001
**Use of mosquito coils**	240(92.0, 88.0–95.0)	60(98.4, 91.2–100.0)	80(80.0, 70.8–87.3)	100(100.0, 100.0–100.0)	< 0.0001
**Fogging and spraying with insecticides**	137(52.5, 46.2–58.7)	28(45.9, 33.1–59.2)	18(18.0, 11.0–27.0)	91(91.0, 83.6–95.8)	< 0.0001
**Use of bed nets**	128 (49.0, 42.8–55.3)	9(14.8, 7.0–26.2)	29(29.0, 20.4–38.9)	90(90.0, 82.4–95.1)	< 0.0001
**Others**	11(4.2, 2.1–7.4)	1(1.6, 0.04–8.8)	9(9.0, 4.2–16.4)	1(1.0, 0–5.5)	0.0099
**Not know or no response**	10(3.8, 1.9–6.7)	0(0, 0–5.9)	10 (10.0, 4.9–17.6)	0(0, 0–3.6)	< 0.0001
**Family income source**	*n* = 260				
**Agriculture**	123 (41.1, 42.8–53.6)	1(1.6, 0.04–8.8)	31(31.3, 22.4–41.4)	91(91.0, 83.6–95.8)	< 0.0001
**Others**	137(52.7, 46.4–58.89)	60(98.4, 91.2–100)	68(68.7, 58.6–77.6)	9(9.0, 4.2–16.4)	< 0.0001
**Family decision**	*n* = 261				
**Wife or co-decision**	156 (59.8, 53.5–65.8)	39(63.9, 50.6–75.8)	48(48.0, 37.9–58.2)	69(69.0, 59.0–77.9)	0.0167
**Husband**	105(40.2, 34.2–46.5)	22(36.1, 24.2–49.4)	52(52.0, 41.8–62.1)	31(31.0, 22.3–41.0)	0.0167
**Control Willing**					
**Willing to eliminate bamboo and tree stub holes around houses**	*n* = 226	*n* = 32	*n* = 96	*n* = 98	
	221(97.8, 94.9–99.3)	29(90.7, 75.0–98.0)	96 (100.0, 100.0–100.0)	96(98.0, 92.8–99.8)	–
**Willing to clean dumps and turn containers upside down**	*n* = 239	*n* = 40	*n* = 99	*n* = 100	
	233(97.5, 94.6–99.1)	36(90.0, 76.3–97.2)	99 (100.0, 100.0–100.0)	98(98.0, 93.0–99.8)	0.0027
**Willing to seek treatment first for dengue fever from public health facilities**	96 (36.8, 30.9–42.9)	16 (26.2, 15.8–39.1)	46 (46.0, 36.0–56.3)	34 (34.0, 24.8–44.2)	0.5436

^a^*95%CI* 95% confidence interval; for all variables, total 261 respondents (61 from Mandou, 100 Mandahuo and 100 Jingmenghun), unless otherwise indicated. Not all subtotals add up to the total of 261 owing to missing values

### Health themes identified from qualitative study

All participants of the qualitativ**e** study were Dai people. The analysis of qualitative data identified five health themes: 1) Differences of key informant’s beliefs existed between the urbanized community (Manduo) and the two rural communities (Mandahuo and Jingmenghun). All key informants believed keeping living and working areas clean to benefit for their health. However, the six key informants from the urbanized community did not believe that the Lord Buddha could bless and protect good people from diseases despite that five of them were Buddhist. In contrast, the 12 key informants of the two rural communities believed the Lord Buddha could protect them from diseases if they performed well. The six key informants from the urbanized community denied any connections between diseases and economic conditions, but they believed that human health was associated with natural environment. The 12 key informants of the two rural communities believed that both economic conditions and natural environment were associated with their health. 2) Key informants believed that most of their villagers had sound knowledge on the symptoms, vector and transmission of dengue fever. The key informants in the two communities with locally infected dengue fever cases (Manduo and Mandahuo) knew more about dengue fever than the key informants in the community with only one imported dengue case (Jingmenghun) in 2017. Their distinct knowledge gap between two kinds of the communities with locally infected dengue fever cases and the community with only one imported dengue case was about *Aedes spp* and *Aedes* larvae breeding sites, clinical symptoms, causes of dengue fever and dengue prevention. 3) Most of the key informants perceived dengue fever as a severe disease that could take away people’s lives. 4) Key informants perceived that the governmental requirement of social mobilization worked to clear the habitat of *Aedes sp* larva. 5) Most of the key informants perceived that Dai people were willing to seek treatment first from public health facilities if they recognized that they were infected with dengue virus.

### Health perceptions in general

As shown in Table 2, most of the participants (70.0%, 182/260) believed that the Lord Buddha would protect good people. A significantly lower proportion of the participants (5%, 3/60) in the urbanized community had the belief that the Lord Buddha would protect good people compared to the participants in the two rural communities (100%, 99/99 in Mandahu and 80%, 80/100 in Jingmenghun). During in-depth interview in Mandahuo, one key informant noted “The Buddha will bless and protect good people. If anyone does good things, he or she will get the blessings from the Lord Buddha”. However, only one of the key informants agreed this belief in the urbanized community.

Most of the participants (76.5%, 198/259) agreed that human health were associated with natural factors. A significantly higher proportion of the participants in the urbanized community (100%, 59/59) believed that people’s diseases were related to the poor hygiene compared to those in the two rural communities (80.8%, 80/99 in Mandahuo and 81%, 81/100 in Jingmenghun). During in-depth interview in the urbanized community, one key informant noted “The hotter weather, the more people get ill. There are fewer diseases in cold weather days”; and another one said “The water is very important for human health because people have to drink water every day. The dirty water may cause diseases”. In Jingmenghun, one of the two rural communities, one key informant noted “Fresh air is good for health. The fewer trees lead to the poorer air, and then the poorer air may cause diseases”. As shown in Table 2, only about one-third of the participants (29.7%, 77/259) agreed to that poverty was one of disease causes. A significantly higher proportion of the participants (54.0%, 54/100) in Jingmenghun, one of the two rural communities, agreed to that poverty was one of disease causes compared to those in the urbanized community (11.7%, 7/60) and Mandahuo, another one (16.2%, 16/99) of the two rural communities. In Jingmenghun, one of the key informants noted “The poor may be unhappy, malnutrition and unable to go to see doctors in time”.

### Dengue fever knowledge

As shown in Table 2, most of the participants (88.5%, 231/256) heard about dengue. Many of them (81.4%, 2061/253) were aware that fever was a primary symptom of dengue fever. More than half of the participants (55.3%, 140/253) reported pantalgia. Less than one-third of the participants were aware that orbital pain (25.7%, 65/253) in the eyes and rashes (19.4, 49/253) were ones of dengue fever symptoms. Just one of the key informants did not reported that fever was one of dengue fever symptoms. More than one-third key informants (38.9%, 7/18) did not know more specific symptoms of dengue fever such as pantalgia, rashes and orbital pain in the eyes. Only five of the key informants mentioned that rashes were one of dengue fever symptoms, and two of the key informants mentioned orbital pain in the eyes. About half of the quantitative study participants (50.2%, 130/259) were aware that dengue fever was a severe illness. More than one-third of the participants (39.4%, 102/259) were aware that dengue fever was a deadly disease. A significantly lower percentage of the participants in the urbanized community (26.2%, 16/60) had the perceived severity of dengue fever compared to those in the border community (Mandahuo, 62.0%, 62/100). Most of the key informants (88.9%, 16/18) perceived that dengue fever was a severe or deadly disease. One of the key informants in the urbanized community noted “No deaths occur in Jinghong City, but heard about deaths in Laos and Myanmar. If a dengue fever patient did not receive treatment in time, he or she might become severe or even death”. One of key informants in the border community said “If someone contracted dengue fever for the second time, the disease could be severer than the first attack”. One of key informants in the community with only one imported dengue fever case (Jingmenghun) said “Dengue fever is a severe disease, especially in the first seven days of the illness. Dengue fever is a transmittable disease and it may cause social panic when it becomes epidemic”. However, one of key informants in border community (Mandahu) noted “Dengue fever should not be a dangerous disease in modern medical situation. Dengue fever is curable”. One of key informants in the community with only one imported dengue fever case said “Dengue fever cannot lead to a death. One of my neighbors contracted dengue fever and was cured in the Menghai County Hospital”.

More than three-quarters of the participants (75.9%, 198/259) knew that dengue fever was an infectious disease. A small part of the participants (14.1%, 36/256) thought that dengue fever was able to directly transmit from person to person by speaking, breathing and physical contact. As shown in Table 2, a significantly higher percentage of the participants in the urbanized community (100%, 60/60) knew that dengue fever was transmittable compared to those in the two rural communities (Mandahuo 62%, 62/100 and Jingmenghun 53.1%, 52/99). Many of the participants (82.2%, 213/259) knew that mosquitoes transmitted dengue fever. However, only a few of participants (3.5%, 9/259) knew that a virus caused dengue fever. A significantly higher proportion of participants in the urbanized community (98.4%, 60/61) knew that mosquitoes transmitted dengue fever compared to those in the two rural communities (Mandahuo 70%, 70/100 and Jingmenghun 84.7%, 83/98). Most of the key informants (77.8%, 14/18) confirmed that dengue virus was only transmitted by mosquitoes. However, one of the key informants in the community with only one imported dengue fever case) noted “Toxins cause dengue fever. People would be ill if there are toxins in their bodies. The toxins can be transmitted by physical contacts and breathing”. All six key informants in the border community agreed that in Dai ethnic language dengue fever was called “Paya Yong”, in which “Paya” was illness and “Yong” was mosquitoes. This Dai ethnic name for dengue fever suggested that dengue fever was associate with mosquitoes in Dai ethnic language. However, all 12 key informants in the two non-border communities did not agree that there was a Dai ethnic language name for dengue fever. This may imply that dengue fever in culture is also an imported disease among Dai people in China.

Most of the participants (76.1%, 194/255) knew that dengue vector was *Aedes sp* or piebald mosquitoes. Less than half of the participants (44.8%, 117/261) were aware that *Aedes sp* bit people in daytime. Many of the participants (92.0%, 240/261) reported that use of mosquito coils could effectively prevent bite *of Aedes sp*. Most of the participants (89.1%, 220/260) were aware that *Aedes sp* bred in containers or small-scale pond with clean and stagnant water. A lot of the participants (92.7%, 242/261) agreed that turning containers upside down could reduce number of *Aedes* mosquitoes to prevent dengue fever (Table 2). Similar results were obtained during in-depth interview. One of the key informants noted “Ponded water between houses is easy to breed *Aedes sp* in raining season. However, it is difficult to clear the ponded water”. Most of the key informants (94.4%, 17/18) agreed that breeding site management could reduce number of *Aedes spp* mosquitoes to prevent dengue fever. All six key informants in the urbanized community emphasized environmental management as importance to control of *Aedes spp* mosquitoes compared to those in the two rural communities.

### Control willingness and related factors

As Table 2 shows, most of the participants (97.8%, 221/226) indicated their willingness to eliminate bamboo and tree stub holes around their houses. When all participants were asked if they would consider turning containers upside down, 97.5% (233/239) of them answered “yes”. However, this answer was not fully consistent with results of the qualitative study. Results of the in-depth interview conducted in the urbanized community indicated that the local government has enforced environmental management to control *Aedes* mosquitoes. A village leader introduced “The terms of ‘The Villager Convention of Manduo’ request all families to clean their houses and surrounding environment every day. The team of village leaders conducts two rounds of supervision-visits every week. If *Aedes sp* larvae were found in a household, the family should pay a fine of CNY500. If *Aedes sp* larvae were found again in the same household, the fine would increase to CNY1000. If any household did not comply with the terms of the Villager Convention, we could stop supply of the pipe water and the electricity”. Results of the in-depth interview conducted in the two rural communities indicated that their fellow villagers did not like to clean and maintain environmental hygiene. One of the key informants in the community with only one imported dengue fever case noted “Some villagers have not recognized the severity of dengue fever. They do not like to clean their houses and surrounding environment. We need health education to promote their knowledge, awareness and action”. Results of multivariate logistic analysis (MLA) showed that the participants with FWI 4–5 [odd ratio (OR) 22.9728, 95% confidence interval (CI): 2.4257–217.5688, *p* = 0.0063] were more likely to turn containers upside down compared to those with FWI 1–3; and the participants in the urban community (OR: 0.0239, CI: 0.0019–0.3032, *p* = 0. 0.0040) were less inclined to turn containers upside down compared to those in the two rural communities (Table 3).

**Table 3:** Factors associated with environmental management willingness for vector control (*N* = 261)

Independent variables	No. respondents of WTCUD^a^ (%, 95 CI)	Crude OR^b^ (95% CI^c^)	***P*** values	Adjusted OR^b^ (95% CI^c^)	***P*** values
**Location of villages**					
**Jinghong (*n*** **= 40)**	36 (90.0, 76.3–97.2)	0.0914 (0.0161–0.5176)	0.0068	0.0239 (0.0019–0.3032)	0.0040
**Menghai (*n*** **= 199)**	197 (99.0, 96.4–99.9)	1		1	
**Sex of participants**					
**Female (*n*** **= 165)**	146 (96.7, 92.4–98.9)	0.3356 (0.0386–2.9202)	0.3226		
**Male (*n*** **= 96)**	87 (98.9, 93.8–100)	1			
**Age of participants (years)**					
**17–40 (*n*** **= 124)**	39 (97.5, 86.8–99.9)	0 (0 - > 1.0E12)	0.9626		
**41–84 (*n*** **= 137)**	127 (100, 97.1–100)	1			
**School education**					
**> 6 (*n*** **= 132)**	36(100, 90.3–100)	0.1852 (0.0213–1.6091)	0.1263	0.4364 (0.0371–5.1366)	0.9963
**≤ 6 (*n*** **= 129)**	121 (99.2, 95.5–100)	1		1	
**Family wealth index**					
**4–5 (*n*** **= 246)**	115 (99.1, 95.3–100)	10.0371 (1.6559–60.8373)	0.0121	22.9728 (2.4257–217.5688)	0.0063
**1–3 (*n*** **= 13)**	11 (84.6, 54.6–98.1)	1		1	
**Belief of Buddha blessing good people**					
**Yes (*n*** **= 181)**	178 (98.3, 95.2–99.7)	3.2364 (0.6353–16.4873)	0.1574	2.8571 (0.4992–16.3514)	0.2382
**No (*n*** **= 58)**	55 (94.8, 85.6–95.9)	1		1	
**Belief of all natural factors influencing human health**					
**Yes (*n*** **= 177)**	171 (96.6, 92.8–98.8)	Undefined OR	0.9620		
**No (*n*** **= 61)**	61 (100.0, 100.0–100.0)	1			
**Belief of sound hygiene helpful to people’s health**					
**Yes (*n*** **= 199)**	194 (97.5, 94.2–99.2)	0.9949 (0.1131–8.7512)	0.9963		
**No (*n*** **= 40)**	39 (97.5, 86.8–99.9)	1			
**Respondents have heard about dengue**					
**Yes (*n*** **= 210)**	204 (97.1, 93.9–98.9)	Undefined OR	0.9739		
**No (*n*** **= 29)**	29 (100.0, 100.0–100.0)	1			
**Perceive dengue as a severe or deadly disease**					
**Yes (*n*** **= 101)**	96 (95.1, 88.8–95.4)	0.1422 (0.0164–1.2360)	0.0771	0.1830 (0.0202–1.6549)	0.1306
**No (*n*** **= 136)**	135(99.3, 96.0–100.0)	1		1	
**Perceived easy to be infected by dengue**					
**Yes (*n*** **= 74)**	71 (96.0, 88.6–99.2)	0.4438 (0.0874–2.2518)	0.3269		
**No (*n*** **= 163)**	160 (98.2, 94.7–99.6)	1			
**Know dengue only transmitted by mosquitoes**					
**Yes (*n*** **= 61)**	60(98.4, 91.3–100.0)	1.7733 (0.2034–15.4582)	0.6041		
**No (*n*** **= 177)**	172(97.2, 93.5–99.1)	1			
**Know dengue mosquitoes biting in daytime or whole day**					
**Yes (*n*** **= 208)**	203(97.6, 94.5–99.2)	1.4000 (0.1579–12.4093)	0.7625		
**No (*n*** **= 30)**	29(96.7, 82.8–99.9)	1			
**Know at least one breeding site of** ***Aedes spp*** **larvae**					
**Yes (*n*** **= 208)**	203(97.6, 94.5–99.2)	Undefined OR	0.9678		
**No (*n*** **= 27)**	27 (100.0, 100.0–100.0)	1			
**Know at least one method of mosquito adult control**					
**Yes (*n*** **= 83)**	82 (98.8, 93.5–100.0)	Undefined OR	0.9711		
**No (*n*** **= 10)**	10 (100.0, 100.0–100.0)	1			
**Know at least one method of mosquito larva control**					
**Yes (*n*** **= 128)**	122 (95.3, 90.2–98.3)	Undefined OR	0.9695		
**No (*n*** **= 7)**	7(100.0, 100.0–100.0)	1			

^a^*WTCUD* Willing to turn containers upside down, ^b^*OR* Odds ratio, ^c^*95%CI* 95% confidence interval. For all variables, total 261 respondents, unless otherwise indicated. Not all subtotals add up to the total of 261 owing to missing values

When asked where they or their family members would consider to seek treatment if they had an attack of dengue fever, nearly two-third of participants (63.2%, 165/239) reported that they would buy drugs from drugstores first, only about one-third of them (36.8%, 96/239) answered they would go to a public health facility first (Table 2). Results of the qualitative data analysis indicated that Dai people liked to take over-the-counter (OCT) drugs for disease treatment first. The qualitative data analysis summarized out three reasons for this treatment-seeking intention: 1) High accessibility of OCT drugs, one of the key informants said “There are a lot of drugstores anywhere, from the city to the countryside. We can buy most of drugs that we need at any time. The prices of store drugs are much cheaper than hospital drugs”. 2) A long time needs to seek treatment from public hospitals, one of the key informants noted “Travel needs time; waiting to see a doctor needs time”. 3) They have to pay for some unnecessary laboratory tests or physical examinations, one of the key informants noted “The hospital will request us to do a set of laboratory tests or physical examinations. Some of laboratory tests or physical examinations are not actually essential, but we still have to pay for the unnecessary laboratory tests or physical examinations, in spite of Xin Nong He (new rural health insurance in China) can cover parts of the money”. In the MLA, the participants with the perceived severity of dengue fever (OR: 5.0564, CI: 2.0672–12.3683, *p* = 0. 0.0004) compared to those without the perceived severity and the participants with the awareness of sound hygiene (OR: 11.5671, CI: 2.0672–12.3683, *p* = 0. 0.0055) compared to those without awareness of sound hygiene tended to seek treatment first from public health facilities. Suspected dengue fever patients from households with wife or co-decision (OR: 11.5671, CI: 2.0672–12.3683, *p* = 0. 0.0055) were less likely to seek treatment first from public health facilities compared to those in households with husband decision (Table 4).

**Table 4:** Factors associated with seeking treatment first for dengue fever from public health facilities (*N* = 261)

Independent variables	No. respondents of WSTPHF^a^ (%, 95 CI)	Crude OR^b^ (95% CI^c^)	***P*** values	Adjusted OR^b^ (95% CI^c^)	***P*** values
**Location of villages**					
**Jinghong (*n*** **= 61)**	16 (26.2, 15.8–39.1)	0.5335 (0.2822–1.0085)	0.0531	0.8816 (0.1977–3.931)	0.8688
**Menghai (*n*** **= 200)**	807 (40.0, 33.2–47.2)	1		1	
**Sex of participants**					
**Female (*n*** **= 165)**	67 (40.6, 33.0–48.5)	1.5795 (0.9250–2.6971)	0.0941	1.6107 (0.7911–3.279)	0.1889
**Male (*n*** **= 96)**	29 (30.2, 21.3–40.4)	1		1	
**Age of participants (years)**					
**17–40 (*n*** **= 124)**	48 (38.7, 30.1–47.9)	1.1711 (0.7076–1.9380)	0.5390		
**41–84 (*n*** **= 137)**	48 (35.0, 27.1–43.7)	1			
**School education**					
**> 6 (*n*** **= 132)**	54 (40.9, 32.4–49.8)	1.3378 (0.7470–2.3959)	0.3277		
**≤ 6 (*n*** **= 129)**	42 (32.6, 24.6–41.5)	1			
**Family wealth index**					
**4–5 (*n*** **= 246)**	90 (36.6, 30.6–42.9)	0.6731 (0.2194–2.0646)	0.4888		
**1–3 (*n*** **= 13)**	100 (100, 75.3–100)	1			
**Belief of Buddha blessing good people**					
**Yes (*n*** **= 182)**	73 (40.1, 32.9–47.6)	1.6014 (0.9058–2.8313)	0.1053	0.9937 (0.2965–3.3300)	0.9918
**No (*n*** **= 78)**	23 (29.5, 19.7–40.9)	1		1	
**Belief of all natural factors influencing human health**					
**Yes (*n*** **= 198)**	66 (33.8, 27.3–40.9)	0.5644(0.3152–1.0104)	0.9620		
**No (*n*** **= 61)**	29 (47.5, 34.6–60.7)	1			
**Belief of sound hygiene helpful to people’s health**					
**Yes (*n*** **= 210)**	89 (42.4, 35.6–49.4)	3.778 (1.5204–9.3888)	0.0042	11.5671 (2.0505–65.2502)	0.0055
**No (*n*** **= 50)**	7 (14.0, 5.8–26.7)	1		1	
**Heard about dengue**					
**Yes (*n*** **= 231)**	90 (39.0, 32.6–45.6)	2.5532 (1.0045–6.4896)	0.0489	1.2562 (0.2348–6.7201)	0.7898
**No (*n*** **= 30)**	6 (20.0, 7.7–38.6)	1		1	
**Perceive dengue as a severe disease**					
**Yes (*n*** **= 130)**	67 (51.5, 42.6–60.4)	4.0176 (2.3270–6.9366)	< 0.0001	5.0564 (2.0672–12.3683)	0.0004
**No (*n*** **= 129)**	27(20.9, 14.3–29.0)	1		1	
**Know fever as one of dengue symptoms**					
**Yes (*n*** **= 206)**	83 (40.3, 33.5–47.3)	2.2056 (1.0627–4.5775)	0.0337	1.4204 (0.4018–5.0219)	0.5860
**No (*n*** **= 46)**	11 (23.9, 12.6–38.8)	1		1	
**Perceive dengue transmissible**					
**Yes (*n*** **= 198)**	76 (38.4, 31.6–45.5)	1.3393 (0.7329–2.4474)	0.3422		
**No (*n*** **= 63)**	20 (31.8, 20.6–44.7)	1			
**Family income source**					
**Agriculture (*n*** **= 123)**	45(36.6, 28.1–45.8)	1.0038 (0.6055–1.6643)	0.9881		
**Others (*n*** **= 137)**	50(36.5, 28.4–45.2)	1			
**Family decision**					
**Wife or co-decision (*n*** **= 156)**	45(28.9, 21.9–36.6)	0.4293 (0.2562–0.7193)	0.0013	0.3505 (0.1682–0.7305)	0.0051
**Husband (*n*** **= 105)**	51 (48.6, 38.7–58.5)	1		1	

^a^*WSTPHF* Willing to seek treatment first from public health facilities, ^b^*OR* Odds ratio, ^c^*95%CI* 95% confidence interval. For all variables, total 261 respondents, unless otherwise indicated. Not all subtotals add up to the total of 261 owing to missing values

## Discussion

### Health perceptions, dengue knowledge and control willingness

The Shan people in Myanmar and the Dai people in China are the same ethnicity. Just like the Shan people in Myanmar [8], vast majority of Dai people are Buddhists. Most of the participants in the two rural communities (Mandahu and Jingmenghun) believed that good deeds come back to help themselves, and evil deeds haunt themselves. In spite of vast majority of the participants in the urbanized community (Manduo) were Buddhists, they did not believed that the Lord Buddha would protect good people any more. Instead, a significantly higher proportion of the participants in the urbanized community perceived that people’s diseases were associated with the poor hygiene and natural factors compared to those participants in the two rural communities. Taken overall, knowledge of dengue fever was sound among the Dai people. As shown in the investigations of the Shan people [20] and the Kachin people [21] in Myanmar, knowledge level of dengue fever are associated with dengue fever incidence. In this study, the knowledge level of dengue fever in the two communities with dengue fever outbreaks (Manduo and Mandahuo) was significantly higher than the community with only one imported dengue fever case (Jingmenghun) in 2017. The perceived susceptibility and the perceived severity of dengue fever were low among the Dai people. Only a half of the participants perceived dengue fever as a severe disease, and less than one-third of them agreed that they were easy to contract dengue fever. A significantly lower percentage of the participants in the urbanized community had the perceived susceptibility and the perceived severity of dengue fever compared to those in the two rural communities. The participants reported a high willingness of vector control, with vast majority of the participants expressing willingness to eliminate bamboo and tree stub holes, and turning containers upside down. However, a significantly lower percentage of the participants considered to seek treatment first for dengue fever from public facilities. In current study, a significantly lower proportion of the participants in the urbanized community expressed the willingness of vector control and treatment-seeking willingness from public facilities for dengue fever compared to those in the two rural communities. The basic concepts of the HBM are perceived susceptibility, perceived severity, perceived benefits, perceived barriers to taking health action, cues to action and self-efficacy [16, 17]. Wong et al. reported that some heath beliefs could help to prevent dengue fever [20]. An investigation in Cambodia argued that knowledge was unlikely to have any significant effect on practices in its setting [11]. These concepts interpreted why the participants in the urbanized community had a higher knowledge level of dengue fever, but a significantly lower proportion of dengue control willingness compared to those in the two rural communities. Traditional beliefs of the Dai people have changed with the urbanization, and the improved medication has reduced their perceived susceptibility and severity to dengue fever in China. Dengue fever control at the outskirts of cities is a great challenge in the world [10, 22]. Just like the urbanized community of this study, outbreaks of dengue fever were more frequently occurred in cities in China [4–6]. The results of this study indicated the association between dengue control willingness, health belief, perceived susceptibility and perceived severity. The enforced environmental management has been used to control *Aedes spp* in the three study sites, however, the intervention efficacy seems limited. Thus, the Dai people still need more effective health education interventions about the susceptibility, severity and control willingness of dengue fever.

### Barriers of vector control willingness

The breeding sites of *Aedes spp* mosquitos are small water ponds, containers and discarded tires with clean and stagnant water. Living environmental management of *Aedes spp* mosquitoes is thereby becoming the most critical strategy for dengue vector control [10, 23]. Elimination of *Aedes spp* habitats at residents’ houses and yards needs the community participation. The HBM regards that health-related action depends upon the simultaneous occurrence of three categories of factors, i.e. sufficient motivation, perceived threat and cost-beneficial [16]. In this study, the results of MLA indicated that the participants with FWI 4–5 were more likely to turn containers upside down compared to those with FWI 1–3. This might be due to the fact that the wealthier participants had a better understanding of knowledge provided about environmental management to prevent dengue fever. Just like the discussion above, changed health beliefs and reduced perception of the severity and the susceptibility of dengue fever might be the reason why the participants in the urban community were less inclined to turn containers upside down compared to participants in the two rural communities. The participants in the urban community had the highest level of dengue knowledge among the three study sites, however, they had the lowest willingness of vector control. This might be the fact that they have become ennui to the frequent request of enforced environmental management. This finding reveals that more effective health education interventions are essential to promote the Dai people’s perceived severity, perceived susceptibility of dengue fever, and sufficient motivation of vector control.

### Barriers of appropriately treatment-seeking willingness for dengue fever

Appropriately treatment-seeking willingness is essential for surveillance and response of infectious diseases. Suspected dengue patients have intention to seek proper medical care early can reduce the complication of dengue fever, and further reduce transmission of dengue virus [24, 25]. Due to the fact that laboratory test for dengue fever is only available in the public health facilities in China, it is expected that suspected dengue patients should seek treatment first from the public health facilities. Results of the quantitative study indicated that just about one-third of the participants expressed their willingness to seek treatment first for dengue fever from the public health facilities. The MLA identified that the participants with the perceived severity and the participants with the awareness of sound hygiene tended to seek treatment first from public health facilities compared to participants without the perceived severity and the awareness of sound hygiene. This might be the fact that the participants with the perceived severity and the awareness of sound hygiene had a better understanding of information provided about availability of laboratory test for dengue fever in the public health facilities, and the importance of prompt diagnosis and early treatment to relieve symptoms of dengue fever and to reduce related complication. This study suggests that availability of laboratory test for dengue fever in the public health facilities should be focus information to the public. It may be, therefore, worthwhile trialing targeted educational intervention to promote the Dai people’s awareness of the severity and knowledge about dengue fever. The reason why a significantly lower proportion of the participants from the households with wife or co-decision had intention to seek treatment first for dengue fever from the public health facilities compared to those participants from the households with husband decision, is not clear, thus, future investigation should be essential. Findings of the qualitative study revealed that the health authority needs to address a number of important issues on the Dai people’s treatment-seeking willingness, including overuse of OCT drugs, accessibility of laboratory diagnosis, time cost and financial expenditure of diagnosis and treatment service for dengue fever in the public health facilities.

### Challenges of dengue fever control

In Yunnan, the first locally infected dengue fever case was confirmed in 2013 [6], shortly after the successful control of malaria along China-Myanmar border [26]. The pattern of “big year” and “small year” of dengue fever incidence appeared in Yunnan since 2013. The “big year” means a lot number of dengue fever outbreaks in a year, and the “small year” means few outbreaks with a few dengue fever cases within a year. The year after a “big year” was often a “small year” and a “big year” would be found subsequently after a “small year” [27]. Why this phenomenon of “big year” and “small year” for dengue fever appeared is still unknown. The intervention intensity and the local people’s control willingness might be an explanation for this phenomenon. The asymptomatic dengue virus infection was estimated accounting for more than 75% of overall dengue virus infection in the world [28, 29]. In China, when an outbreak happens, the environmental management for controlling *Aedes spp* mosquitoes was enforced by social mobilization and governmental regulations. However, the intervention intensity is usually reduced when without outbreaks and with reduced number of dengue fever cases. The phylogenetic study revealed the high virus relatedness between dengue outbreaks and imported dengue virus in Yunnan [7]. Year 2017 was a “big year” with many breaks, and 2018 was a “small year” without any outbreak, and then 2019 was a “big year” with many outbreaks again. No any outbreak was found in 2020, and only a few of dengue fever cases was detected in the first half year of 2021 due to border limitation for COVID-2019 control. This reveals that dengue fever situation in Yunnan each year may be associated with imported dengue virus and intervention intensity. This finding suggests that people’s control willingness could help to reshape focus messages and approach of health education interventions.

### Limitations

The primary limitation is that this study is quantitatively dominated, using qualitative data to triangulate the quantitative results and to explore reasons for the quantitative results. The depth off qualitative study is not enough to explore causes of existing health perceptions, dengue knowledge, and control willingness. Among the second limitation of this study, a lower response rate of the participants to the questions of willingness in the urbanized community might lead to information bias.

## Conclusion

In conclusion, the health perceptions of Dai people are changing with ongoing urbanization. Most Dai people have sound knowledge, but a low proportion of the participants with the perceived susceptibility and the severity of dengue fever. The willingness of the Dai people in environmental management for vector control and seeking appropriate treatment for dengue fever are two main challenges in dengue fever control. This suggests the need to provide information of laboratory test availability in the public health facilities, and health educational interventions should target to promote the perceived susceptibility and the perceived severity of dengue fever among the Dai people.

## Supplementary Information

**Additional file 1.**

**Additional file 2.**

## Data Availability

All relevant data are within the manuscript. The datasets are available from the corresponding author on reasonable request.

## Abbreviations

WHO: World Health Organization
HBM: Health belief model
TCUD: Turn containers upside down
WTCUD: Willing to turn containers upside down
WSTPHF: Willing to seek treatment first from public health facilities
OCT: Over-the-counter
95% CI: Confidence interval
OR: Odds ratio
FWI: Family wealth index
